# Ultra-Rapid Glutathionylation of Ribonuclease: Is This the Real Incipit of Its Oxidative Folding?

**DOI:** 10.3390/ijms20215440

**Published:** 2019-10-31

**Authors:** Alessio Bocedi, Giada Cattani, Giorgia Gambardella, Silvia Ticconi, Flora Cozzolino, Ornella Di Fusco, Piero Pucci, Giorgio Ricci

**Affiliations:** 1 Department of Chemical Sciences and Technologies, University of Rome “Tor Vergata”, 00133 Rome, Italy; bcdlss01@uniroma2.it (A.B.); giada.cattani@gmail.com (G.C.); giorgia.gambardella@gmail.com (G.G.); silviaticconi@live.com (S.T.); 2 CEINGE Biotecnologie Avanzate and Department of Chemical Science, University of Naples “Federico II”, 80126 Naples, Italy; cozzolinof@ceinge.unina.it (F.C.); difusco@ceinge.unina.it (O.D.F.); pucci@unina.it (P.P.)

**Keywords:** ribonuclease, glutathionylation, oxidative folding, molten globule, cysteine reactivity

## Abstract

Many details of oxidative folding of proteins remain obscure, in particular, the role of oxidized glutathione (GSSG). This study reveals some unknown aspects. When a reduced ribonuclease A refolds in the presence of GSSG, most of its eight cysteines accomplish a very fast glutathionylation. In particular, one single cysteine, identified as Cys95 by mass spectrometry, displays 3600 times higher reactivity when compared with an unperturbed protein cysteine. Furthermore, the other five cysteines show 40–50 times higher reactivity toward GSSG. This phenomenon is partially due to a low p*K*_a_ value of most of these cysteines (average p*K*_a_ = 7.9), but the occurrence of a reversible GSSG-ribonuclease complex (*K*_D_ = 0.12 mM) is reasonably responsible for the extraordinary hyper-reactivity of Cys95. Neither hyper-reactivity nor some protein-disulfide complexes have been found by reacting a reduced ribonuclease with other natural disulfides i.e., cystine, cystamine, and homocystine. Hyper-reactivity of all cysteines was observed toward 5,5’-dithiobis-(2-nitrobenzoic acid). Given that GSSG is present in high concentrations in the endoplasmic reticulum, this property may shed light on the early step of its oxidative folding. The ultra-rapid glutathionylation of cysteines, only devoted to form disulfides, is a novel property of the molten globule status of the ribonuclease.

## 1. Introduction

The oxidative folding of proteins containing disulfide bridges is a critical process in the pathway leading to native conformations [1]. A precise understanding of this event is not a merely speculative exercise, but the basis to know the origin of pathological misfoldings that cause severe diseases like Alzheimer’s and Parkinson’s diseases [2]. It is well known that a correct formation of structural disulfide bonds is crucial for protein integrity and dysregulation of such disulfides may contribute to the pathogenesis of these diseases [3]. In fact, under appropriate conditions a wide range of disulfide-containing proteins can convert from their normally soluble forms into fibrillar aggregates with all the characteristics of disease-associated amyloid fibrils [4]. These aggregates are not normally found in biological systems due to mechanisms that inhibit their formation. Furthermore, disulfide bonds have co-evolved with protein sequences to minimize protein misfolding and propensity to form potentially toxic aggregates [5]; and understanding the nature of such protective mechanisms is a crucial step in the development of strategies to prevent these diseases [6]. In this context, the discovery of new kinetic properties of structural cysteines in the nascent structure of a protein may be of paramount interest and it represents the main novelty of the present study.

The oxidative folding of ribonuclease (RNase) has been extensively studied in the last century, starting with the historical study by the Nobel Prize winner, Anfinsen [7]. It is well accepted that, in the presence of glutathione/oxidized glutathione (GSH/GSSG) in a ratio similar to that found in the endoplasmic reticulum, a reduced ribonuclease (rRNase) first forms mixed disulfides with GSSG and then a restricted number of correct and incorrect protein disulfides through a non-random mechanism [8,9]. The final disulfide to be formed involves Cys40 and Cys95, although a small fraction involves Cys62-Cys75 [10,11]. The possible role of protein disulfide isomerase (PDI) as a catalyst in this process is not supported by mass spectrometry (MS) analyses of the S-S containing peptides, because they are equally populated in the presence or absence of this enzyme [8]. Furthermore, there is strong evidence for endoplasmic reticulum oxidase (Ero1)-independent mechanisms that lead to the production of the oxidized enzyme [12]. In the early stage of the folding process, Cys110, present in many proteolytic fragments containing native and incorrect disulfides, appears to be one of the most reactive residues, while Cys26 is the most inert among the eight cysteines. By observing the kinetics of disulfide formation at pH 8.0, approximate values have been calculated for the formation of the first two disulfides (about 115 M^−1^ s^−1^) [13], a value more than ten-fold higher than those found in a previous paper [14] under similar conditions.

Despite these and many other studies that have been made to detail this process, some important particulars remain to be defined. For example, the intrinsic reactivity of each cysteine in the reduced enzyme has been never evaluated as well as no comparison has been made with the reactivity of a free cysteine. Thus, no evidence has been obtained in the past for the possible existence of cysteines hyper-reactive toward GSSG, which may react with this disulfide in a very short time without the assistance of an enzyme. This reaction may be important to drive a correct oxidative folding. The occurrence of non-functional hyper-reactive cysteines was only rarely investigated in the past for other proteins. In the literature, bovine pancreatic trypsin inhibitor (BPTI) is one of the most cited case of hyper-reactive cysteines intended to form disulfides in the native protein [15]. However, it was recently demonstrated that among its six cysteines, four of them display only about ten times higher reactivity than an unperturbed protein cysteine, while the remaining cysteines are even less reactive [16]. Conversely, we found that Cys94 in reduced lysozyme [17] and Cys75 in reduced serum albumin [16] display thousand times higher reactivity toward GSSG. In the case of lysozyme, glutathionylation of Cys94 causes the instantaneous inhibition of the deleterious aggregation that occurs when lysozyme is in a reduced form [17], giving a reasonable finality for this phenomenon.

This paper discovers that the rRNase in a molten globule like conformation, representing the early proof of its native conformation, shows one particular cysteine hyper-reactive toward GSSG, which is estimated to be thousand times more reactive than an unperturbed protein cysteine. Our study identifies this particular residue and explores the origin of this phenomenon that is in part due to a low p*K*_a_ of this cysteine, but mainly to the rapid formation of a productive protein-GSSG complex.

## 2. Results

### 2.1. Reaction of rRNase with DTNB

**Figure 1:**
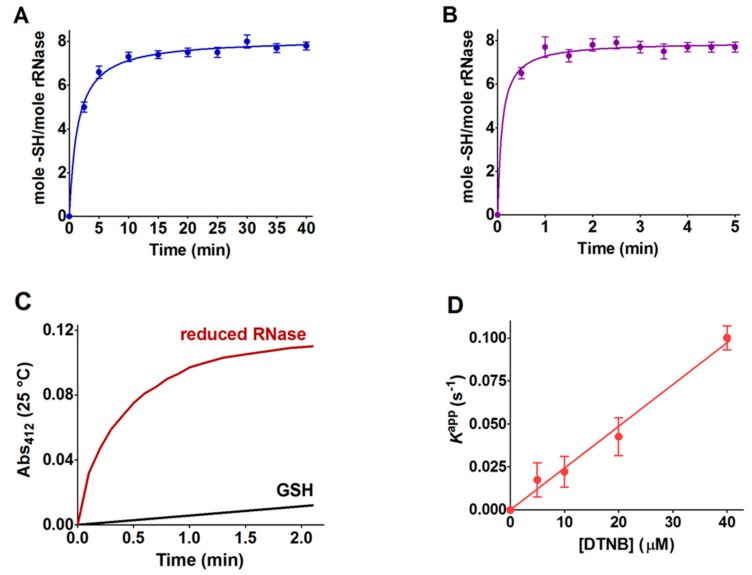
Reduction of the RNase and reaction of the rRNase with DTNB. The reduction of the RNase (0.05 mM) was performed in 10 mM borate buffer, pH 8.5, with 0.5 mM DTT and urea 8 M at two different temperatures 37 °C (**A**) and 60 °C (**B**) (fit obtained using Equation (1)). The reduced cysteines were titrated at different times as reported under Methods. Average ± SD derived from ten independent experiments. (**C**) 1.25 nmol of the rRNase (about 10 nmol titratable cysteines) was reacted with 20 nmol DTNB in 1 mL of 0.1 M sodium acetate buffer, pH 5.0 and 0.2 M urea (red line). For comparison, an identical reaction was performed with 10 nmol of GSH (black line). (**D**) First-order kinetic constants for the reaction of the eight cysteines of the rRNase with different DTNB concentrations at pH 5.0 and 0.2 M urea. Average ± SD derived from three independent experiments.

**Figure 2:**
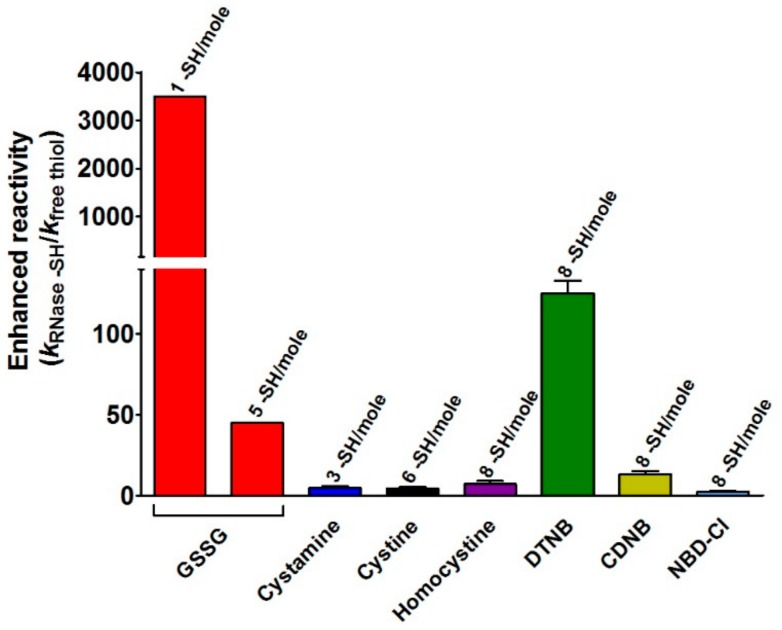
Reactivity of protein cysteines toward disulfides and thiol reagents normalized to that of GSH and free Cys. Second-order kinetic constants of the rRNase toward GSSG (red bars) normalized to the constant calculated for an unperturbed protein cysteine (0.2 M^−1^ s^−1^) (see Materials and Methods) represent the “enhanced reactivity”. All other bars represent the second-order kinetic constants of the rRNase in its reactions with other disulfides and thiol reagents normalized to the constants for free GSH. Error bars represent the propagation of uncertainties for the quotients [20] (see Materials and Methods).

RNase can be efficiently reduced in the presence of only ten molar excesses of dithiothreitol (DTT) (two excess compared to the protein disulfides) and 8 M urea. Figure 1A,B shows the kinetics of reduction at two different temperatures. The reaction was almost completed in about 5 min at 60 °C (7.8 ± 0.3 –SH/mole rRNase), while a very similar reduction was obtained after thirty minutes at 37°C. Both the reducing procedures avoided the use of a large amount of reducing compounds as used in the previous studies. When the reduced enzyme, purified from DTT through Sephadex G-25 chromatography, was incubated with 5,5’-dithiobis-(2-nitrobenzoic acid) (DTNB), a well-known organic disulfide used as a thiol titrant, all of its eight cysteines reacted 130 times faster than a free thiol i.e., GSH (Figure 1C, Table 1, and Figure 2). The reaction appeared to be monophasic and could be followed continuously by monitoring the increase in absorbance at 412 nm due to the release of 5-thio-2-nitrobenzoate (TNBS^–^). The experiment was performed at pH 5.0, because at higher pH values, the reaction with DTNB was too fast to be followed with a usual spectrophotometric apparatus. We also observed a linear dependence of the first-order kinetic constants on the DTNB concentrations (Figure 1D), which excluded the rate-limiting formation of an rRNase-DTNB complex. This unexpected hyper-reactivity was used successfully to follow the disappearance of the reduced cysteines of the RNase due to an oxidation/glutathionylation event without removing residual DTT as it gives a kinetically negligible reaction with DTNB at pH 5.0.

**Figure 3:**
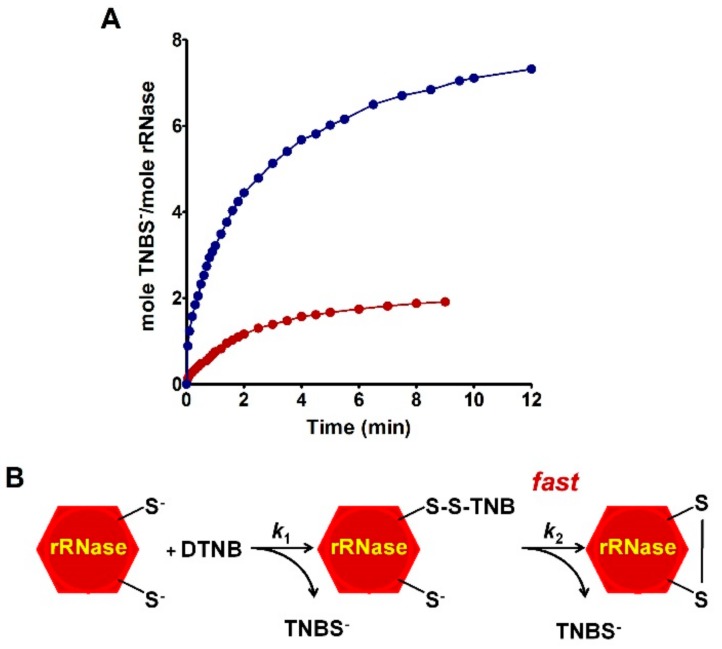
TNBS^–^ release after reaction of the rRNase with substoichiometric DTNB. (**A**) Fully reduced rRNase (1.25 µM, 10 µM protein –SH) was reacted with 1.25 µM DTNB at pH 5.0, 25 °C (red line); the rRNase (1.25 µM, 10 µM protein –SH) was reacted with 5.0 µM DTNB at pH 5.0, 25 °C (blue line). (**B**) Schematic representation of the reaction of the rRNase with stoichiometric DTNB.

Similar experiments with DTNB were always performed at pH 5.0, but with substoichiometric amounts of this reagent (one mole protein (8 –SH group) with one mole DTNB and one mole protein with four moles of DTNB) gave further interesting information. In fact, the amount of TNBS^–^ released during this interaction, proved that each initial mixed disulfide protein-S-S-TNB rapidly evolved toward the formation of protein disulfides (Figure 3A,B).

### 2.2. pK_a_ Determination

**Figure 4:**
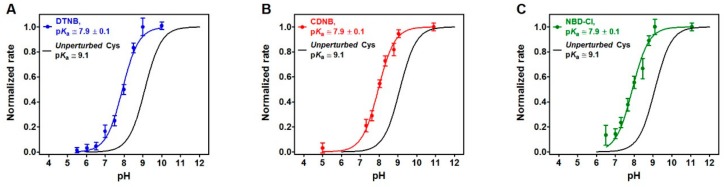
p*K*_a_ determination. The rRNase (1.25 µM) was reacted with 20 µM of DTNB, CDNB, and NBD-Cl at variable pH values as reported under Materials and Methods. Average p*K*_a_ = 7.9 ± 0.1 of the eight reactive cysteines in the rRNase was calculated using (**A**) DTNB, (**B**) CDNB, and (**C**) NBD-Cl (all curves were obtained by fitting to equation (2)). The theoretical curve of an unperturbed protein cysteine (p*K*_a_ = 9.1) is reported in the three panels (black line). Data are the average ± SD of three independent experiments. Differences between p*K*_a_ values were considered statistically significant by *t*-test (*p* < 0.01).

By observing the dependence of the rate of reaction of the rRNase with DTNB at different pH values and using a stopped flow apparatus, we measured an average p*K*_a_ of 7.9 ± 0.1 (Figure 4A) which might be crucial for the observed hyper-reactivity above described and shown in Table 1 and Figure 2. By using two other organic thiol reagents, namely, 1-chloro-2,4-dinitrobenzene (CDNB) and 4-chloro-7-nitrobenzofurazane (NBD-Cl), we measured an identical p*K*_a_ value of 7.9 ± 0.1, a value 1.2 units lower than that of an unperturbed protein cysteine (p*K*_a_ = 9.1) (Figure 4B,C).

### 2.3. Reaction of the rRNase with GSSG

**Figure 5:**
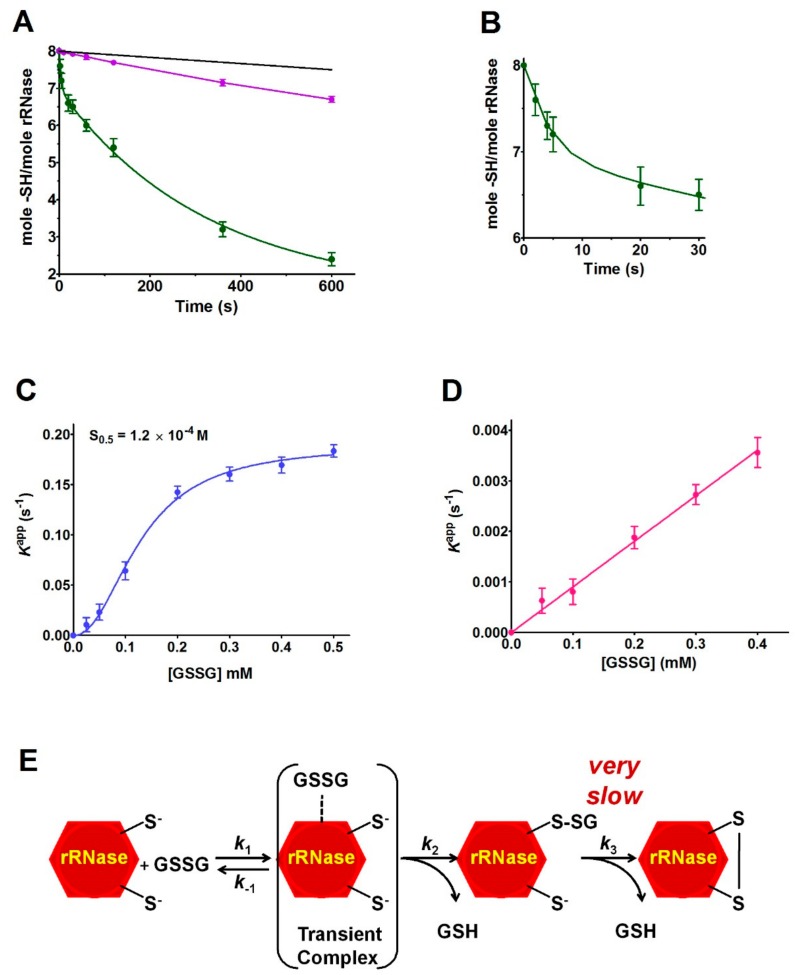
Oxidation of rRNase cysteines by reaction with GSSG. (**A**) The disappearance of rRNase cysteines (1.25 µM, 10 µM in –SH groups) during the reaction with 0.4 mM GSSG at pH 7.4, 0.2 M urea at 25 °C (green line). The reaction was followed by determining residual protein sulfhydryls with DTNB (see Materials and Methods). For comparison, the disappearance of the sulfhydryl group of 10 µM cysteine with 0.4 mM GSSG at pH 7.4, 0.2 M urea at 25 °C is reported (magenta line). Average ± SD from seven independent experiments. Theoretical sulfhydryl groups’ disappearance of an unperturbed protein cysteine (10 µM) during the reaction with 0.4 mM GSSG (black line) (fit obtained using equation (3)). (**B**) Fast-phase of the reaction between the rRNase and GSSG shown in (**A**). (**C**) Apparent first-order rate constants for the reaction of the rRNase hyper-reactive cysteine with different GSSG concentrations at pH 7.4, 0.2 M urea at 25 °C. Average ± SD from three independent experiments (fit obtained using equation (4)). (**D**) Apparent first-order rate constants for the remaining five reactive cysteines of the rRNase with different GSSG concentrations at pH 7.4, 0.2 M urea at 25 °C. Average ± SD from three independent experiments. (**E**) Representative scheme of the reaction of the rRNase with stoichiometric GSSG.

The rRNase reacts with 0.4 mM GSSG showing particularly biphasic kinetics. The first fast phase involved only one protein cysteine and it ended within a few seconds (t_1/2_ = 3.5 ± 0.4 s), while the second step represented a slow oxidation/glutathionylation of five more cysteines (t_1/2_ = 185 ± 12 s) (Figure 5A,B). The corresponding second-order kinetic constants were 430 M^−1^ s^−1^ for the fast phase and about 9 M^−1^ s^−1^ for the slow phase (Table 1). These values indicated that the first fast and the second slow phases were about 2150 and 45 times faster than an unperturbed protein cysteine (*k* = 0.2 M^−1^ s^−1^) [17] (Figure 2).

By plotting the observed first-order kinetic constants of the fast phase as a function of GSSG concentration, a saturation behavior was clearly observed (Figure 5C), which strongly suggested the involvement of an intermediate reversible (E-GSSG) complex which might justify the extraordinary reactivity toward GSSG shown above. The sigmoidal behavior also indicated a cooperative-like binding with an [S_0.5_] of 0.12 mM (Figure 5C). The second-order kinetic constant of the hyper-reactive cysteine, calculated at 0.1 mM GSSG (a non-saturating concentration), indicated a more correct value of *k* = 700 M^−1^ s^−1^ with an incremental kinetic factor of 3500 when normalized to an unperturbed protein cysteine.

A very similar biphasic kinetic behavior was observed when the reaction of the protein cysteines with GSSG was followed by evaluating the production of free GSH. On the contrary, the observed first-order kinetic constants of the slow phase plotted as a function of GSSG showed a linear behavior suggesting the absence of any rate-determining intermediate complex (Figure 5D). A reaction scheme is reported in Figure 5E.

### 2.4. Reaction of the rRNase with Other Natural Disulfides and Thiol Reagents

The reactions of the rRNase with other natural disulfides like cystine, homocystine, and cystamine and with alkylating reagents like CDNB and NBD-Cl were performed as reported under Materials and Methods.

As shown in Table 1 and Figure 2, only three and six protein cysteines reacted with cystamine and cystine respectively, which are hydrophilic disulfides while all the eight residues were modified by homocystineand by the lipophilic reagents, CDNB and NBD-Cl. For all these reagents, no cysteine hyper-reactivity was found except for a ten-fold increased reactivity for CDNB. In addition, the second-order kinetic constants calculated at different levels of each disulfide do not show any trace of saturation indicating the absence of a protein-disulfide reversible complex, contrary to that observed for GSSG.

### 2.5. The rRNase Conformation Retains a Partial Ordered Structure Even in 8 M Urea

**Figure 6:**
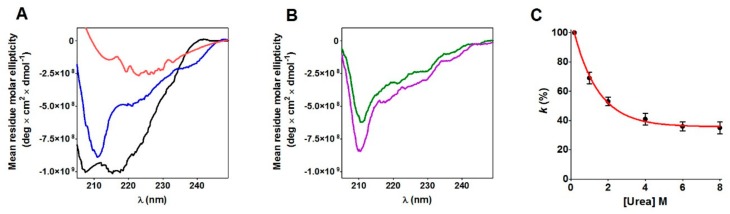
Circular dichroism spectra of the RNase and the effect of urea on the hyper-reactivity of rRNase cysteines. (**A**) CD spectra of native ribonuclease (black line); reduced RNase in 0.2 M urea (blue line); reduced RNase in 8 M urea (red line). CD spectra were recorded at pH 7.4 (see Materials and Methods). (**B**) CD spectra of alkylated rRNase (green line); glutathionylated/alkylated rRNase (magenta line). CD spectra were recorded in 0.2 M urea at pH 7.4 (see Materials and Methods). (**C**) Hyper-reactivity of rRNase cysteines (10 µM) toward DTNB (20 µM) at different urea concentrations (pH 5.0, 25 °C) (fit obtained using equation (5)). Average ± SD from three independent experiments.

The circular dichroism (CD) spectra of the rRNase are reported in Figure 6A. A comparison with the spectrum observed for the native conformation indicated that a relevant amount of secondary structures were conserved at 0.2 M urea—the final concentration used in all kinetic experiments made in this study. Unexpectedly, a residual ordered structure, possibly due to a residual alpha-helix conformation, was still present in 8 M urea. Moreover, the comparison between CD spectra of the rRNase and glutathionylated-rRNase (Figure 6B) indicated that the glutathionylation of a single cysteine promoted a further structuration of the molten globule. In the presence of 8 M urea, the hyper-reactivity toward DTNB was not fully abolished, but about 35% was still present (Figure 6C). On the contrary, in 8 M urea, the hyper-reactivity toward GSSG completely disappeared for both the fast- and slow-reacting cysteines (*k* = 0.8 M^−1^ s^−1^) (Table 1).

### 2.6. Mass Spectrometry Identified Cys95 as the Hyper-Reactive Residue toward GSSG

**Figure 7:**
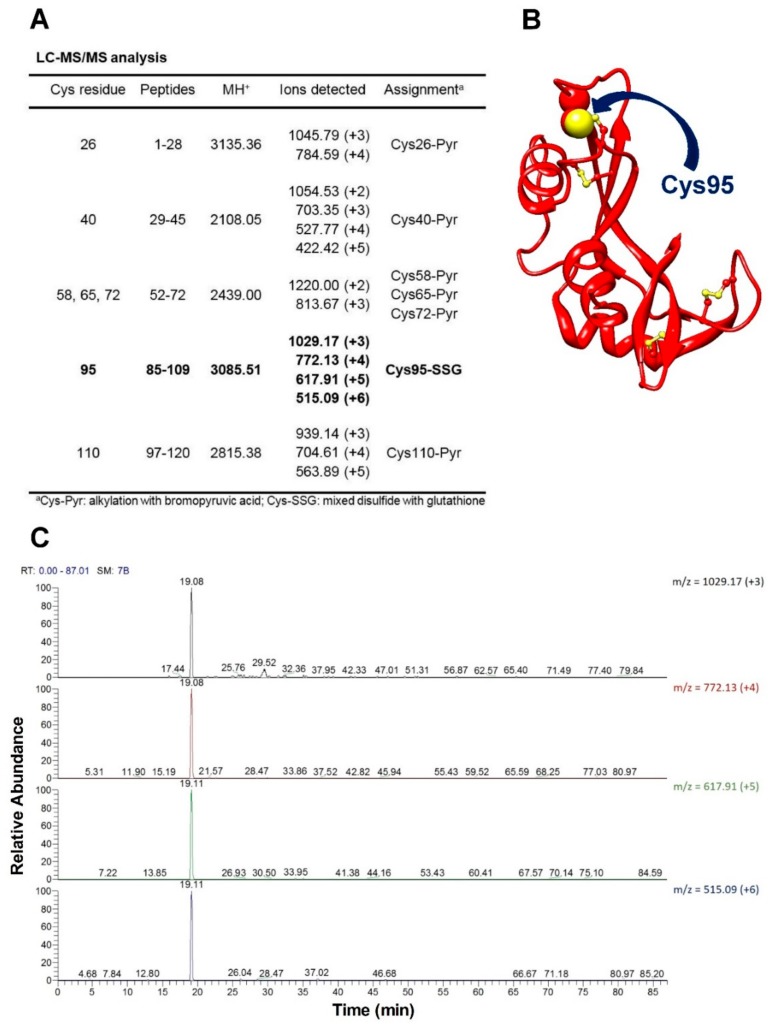
Mass spectrometry identification of the most reactive cysteine of the rRNase toward GSSG. (**A**) Cys-containing peptides identified in the LC-MS/MS analysis of controlled pepsin hydrolysis of the rRNase treated with 0.4 mM GSSG at pH 7.4 for 10 s and then alkylated with 1 mM bromopyruvic acid. (**B**) Three-dimensional structure of native RNase A from bovine pancreas (PDB id: 1FS3) [21] represented in red ribbons; cysteines are in yellow ball-and-stick, the most hyper-reactive cysteine (Cys95) is highlighted by a yellow sphere and indicated by the blue arrow. The picture was drawn by using UCSF Chimera [22]. (**C**) Ion-extraction chromatograms of the tri-, four, five-, and six-fold charged ions corresponding to the 85–109 peptide containing the glutathionylated Cys95.

The rRNase was reacted with 0.4 mM GSSG at pH 7.4 for only ten seconds and the sample was then treated with 1 mM bromopyruvic acid to alkylate the residual protein cysteines within 1–2 s. A second rRNase sample was alkylated with bromopyruvate and used as a control. The reaction was stopped by lowering the pH to 5.0, desalted by reversed-phase HPLC, and digested with pepsin under controlled conditions as described in the Materials and Methods section. The resulting peptides were analyzed by nanoLC-MS/MS. Peptides were mapped onto the anticipated RNase amino acid sequence leading to the identification of Cys-containing fragments. All cysteines were found modified by pyruvic acid, except Cys95 (Figure 7A,B). Figure 7C shows the ion extraction chromatograms of the tri-, four-, five-, and six-fold charged ions corresponding to the 85–109 peptide containing the glutathionylated Cys95. The assignment was confirmed by manual inspection of the corresponding fragmentation spectrum.

## 3. Discussion

This study discloses for the first time the existence, in the rRNase, of a curious and extraordinary hyper-reactivity of one cysteine toward GSSG and a parallel relevant hyper-reactivity of five other cysteines (3500 and 45 times more reactive when compared to an unperturbed protein cysteine, respectively) (Table 1, Figure 2 and Figure 5).

Hyper-reactivity toward GSSG was previously demonstrated for Cys75 in reduced albumin [16] and then for Cys94 in reduced lysozyme [17] (*k* > 250 M^−1^ s^−1^ and *k* = 600 M^−1^ s^−1^, respectively). These values, normalized to that of an unperturbed protein cysteine i.e., 0.2 M^−1^ s^−1^, fulfill enhanced reactivity > 1250 times and 3000 times for Cys75 and Cys94, respectively.

The novelty of the present study is that all the previous investigations about the oxidative folding of rRNase did not evaluate the intrinsic reactivity of the involved cysteines and no comparison has been made with the reactivity of a free cysteine or an unperturbed protein cysteine. Thus, our findings disclose a fascinating aspect, still now completely unknown, about a protein whose oxidative folding probably has been the most studied in the last century. The most hyper-reactive cysteine in the rRNase was identified as Cys95 by mass spectrometry, which revealed the presence of the sole Cys95-SG mixed disulfide after only 10 s incubation with 0.4 mM GSSG.

It is generally accepted that the hyper-reactivity of protein cysteines is modulated by two distinct properties: solvent exposure and p*K*_a_ values [23]. A lowered p*K*_a_ from 9.1 to 7.9, as measured for rRNase cysteines using DTNB, CDNB and NBD-Cl as thiol reagents (Figure 4), could account for only about 15 times higher reactivity. The hundred times increased reactivity toward DTNB of all the protein cysteines and the 45 times higher reactivity toward GSSG for the five protein cysteines could only be explained by assuming the existence of a weak electrostatic positive environment near most of the protein cysteines given that DTNB and GSSG are negatively charged disulfides. This possibility may also explain the average lower p*K*_a_ values. However, the extraordinary hyper-reactivity observed for Cys95 toward GSSG requires further assumptions. In fact, in a thiol-disulfide reaction, the enhanced reactivity of a cysteine due to a very low p*K*_a_ of its sulfhydryl group cannot exceed 40 or 50 times at pH 7.4 [17]. A completely novel factor could be the occurrence of a transient protein-GSSG complex which may promote a productive encounter between the –SH group of Cys95 and GSSG, reminiscent of the encounter of two substrates in the active site of an enzyme. The existence of such a transient complex is strongly suggested by the saturation curve shown in Figure 5C. The plot shows an S_0.5_ of 0.12 mM, a value which warrants most of the rRNase to be “saturated” by GSSG at the physiological concentrations of this disulfide in the endoplasmic compartment (0.4 mM), and then to be glutathionylated at the maximal velocity. The cooperative-like behavior shown in Figure 5C cannot be easily explained. In fact, the RNase is not a multimeric enzyme so the autocatalytic behavior can be tentatively justified assuming that the molten globule displays two binding sites for GSSG (one of them modulating the affinity of the other), or a transient assembly of two enzyme monomers to give a dimeric structure. Interestingly, it was demonstrated that cooperativity requires neither multiple ligand binding events nor multimeric assemblies [24].

The specificity of the above proposed interaction between GSSG and rRNase is indirectly supported by the absence of hyper-reactivity toward other natural disulfides like cystamine, homocystine, and cystine (Table 1) and by the absence of a saturation behavior of the other cysteines in their reaction with GSSG (Figure 5D). All these findings may help to enlighten novel details about the oxidative folding of this enzyme occurring in the cell. In fact, it may be tentatively proposed that the early event of this process may be the glutathionylation of Cys95 possibly finalized to give a hierarchic formation of the natural disulfides restricting the occurrence of incorrect disulfides. This glutathionylated residue could remain as such until all the other native disulfides are formed, as suggested by previous studies indicating the Cys95-Cys40 as the last disulfide in the temporal sequence of the folding [10,11]. This covalent modification of Cys95 could serve to “freeze” this residue to avoid its reaction with other cysteines or, more probably, to induce a useful conformational change. In fact, CD spectra show that the glutathionylation of Cys95 triggers a slight but evident structuration of the enzyme (Figure 6B). The importance of Cys95 and its glutathionylation in the correct folding is indirectly indicated by previous in vivo experiments using a mutated enzyme with Cys95 and its natural counterpart Cys40 replaced by Ala [25]. The consequence of this mutation on the folding mechanism in a living cell was surprising: although these two cysteines are the last residues to form the proper disulfide, their substitution causes a deep change in the oxidative pathway [25]. We can now speculate that glutathionylation of Cys95 and the consequent structural change of the enzyme possibly avoids this non-natural route. Thus, the role may be different from the one suggested for Cys110 which may function as an internal catalyst and is able to promote the reshuffling of disulfides to speed the attainment of the native conformation [26]. Obviously, the proposed role of Cys95 related to its hyper-reactivity toward GSSG must be confirmed by future studies. Unfortunately, the obvious experiment in vivo consisting of the depletion of GSSG by inhibiting its biosynthetic pathway has been done in the past [27], but the presence of relevant levels of oxidized proteins has led to the hasty and perhaps incorrect conclusion that GSSG does not have a role in oxidative folding. In fact, as discussed by Bass and coworkers [28] in the absence of glutathione, all the cell environment becomes strongly oxidant and a secondary oxidative pathway may substitute GSSG in its role.

The presence of one single hyper-reactive cysteine toward GSSG in the rRNase is a property very similar to that reported for the reduced form of lysozyme [17] and serum albumin [16]. This may suggest a more general property of the molten globule conformation of proteins whose cysteines are devoted to structural and not to catalytic roles. This transient and still now unknown structure is far from a disordered or amorphous conformation, but it appears as a more “organized” status able to perform specific functions like to bind GSSG near Cys95 with high affinity. Accordingly, a relevant portion of the ordered structure of the rRNase is present in 0.2 M urea (Figure 6A). The structure of the RNase molten-globule is unknown and the contribution of a docking simulation (based on the native structure) to identify residues involved in the RNase-GSSG complex is clearly limited. However, assuming that the structure of the molten-globule is similar to the one of the native crystal structure [21], a preliminary docking analysis indicates four residues possibly involved in hydrogen bond interactions with GSSG bound near Cys95 (i.e., Lys31, Thr36, Lys37, and Pro93).

While the hyper-reactivity of Cys94 in lysozyme and its fast conversion into a glutathionylated residue seem to be finalized to prevent the deleterious aggregation which occurs when the protein is in a fully reduced status, a similar role cannot be ascribed for rRNase which do not aggregate in such conditions. As proposed above, this particular property may limit the formation of incorrect disulfides in the nascent phase. However, other functions may be taken into account. For example, it was demonstrated that in the human small copper chaperone Cox17, the conformational switch between disordered and folded states is controlled by the formation of a single disulfide bond [29]; a single fast glutathionylation may have a similar role. Finally, we cannot forget the moderate hyper-reactivity found in the remaining cysteines which are greatly more reactive when compared to the cysteines of BPTI, always cited in the literature as a model of hyper-reactivity of structural cysteines [15,16]. Whatever the finality, this phenomenon confirms the presence in the protein science of still new and fascinating mechanisms that may help to clarify more in detail the still obscure event of oxidative folding.

## 4. Materials and Methods

### 4.1. Chemicals and Reagents

Ribonuclease A (RNase) from bovine pancreas (Type XII-A, 75–125 Kunitz units/mg protein) was used. The commercial stock of the RNase used in all the experiments was ≥99% pure according to our HPLC/MS analysis. l-cysteine (Cys), l-cystine, l-glutathione (GSH), oxidized glutathione (GSSG), homocystine, cystamine, 1-chloro-2,4-dinitrobenzene (CDNB), 5,5′-dithiobis(2-nitrobenzoic acid) (DTNB), 4-chloro-7-nitrobenzofurazane (NBD-Cl), dithiothreitol (DTT), ethylendiaminetetraacetic acid (EDTA), bromopyruvic acid, and all other reagents were from Sigma-Aldrich (St. Louis, MO, USA).

### 4.2. RNase Reduction

The RNase concentration was evaluated by an extinction coefficient of 9440 M^−1^ cm^−1^ at 280 nm [30]. The reductions of the RNase at 37 °C and 60 °C were performed as follows: 0.05 mM final concentration in 0.01 M sodium borate buffer, pH 8.5, with 8 M urea, EDTA 1 mM final concentration, and DTT (RNase:DTT, 1:10). The pH was adjusted to 8.5 with 0.1 M NaOH. At fixed times, the content of reduced cysteines was evaluated by reacting the rRNase (1.25 μM) with 20 μM DTNB at pH 5.0 (ε_M_ TNBS^−^ = 11800 M^−1^ cm^−1^ at pH 5.0) and 0.2 M urea taking advantage of the reactivity of all the eight protein cysteines with this reagent (see Table 1). Within the manuscript, the term rRNase refers to that obtained by reduction with DTT after 40 and 5 min at 37 and 60 °C, respectively. DTT stock solutions were titrated with DTNB prior to use.

An alternative procedure was applied for the preparation of the rRNase for experimental measures, with the conditions for the reduction being slightly different. Briefly, 0.1 mM of the RNase was dissolved in 0.01 M sodium borate buffer, pH 8.5, 8 M urea, EDTA 1 mM, and DTT (RNase:DTT, 1:100) at 37 °C for 30 min. After reduction, the rRNase (0.1 mM) was passed through a Sephadex G-25 gravity flow column (1 × 20 cm) equilibrated with 20 mM sodium phosphate buffer, pH 7.4, containing 2 M urea and 1 mM EDTA. The eluted protein in a single fraction (≈40 μM) without DTT was used for the determination of the second-order rate constants, circular dichroism spectra, and p*K*_a_ values.

### 4.3. Reactivity of rRNase Cysteines toward GSSG

The interaction of the rRNase with GSSG (0.4 mM, same concentration of endoplasmic reticulum) [31] was measured by incubating 1.25 μM of protein with GSSG in 0.01 M potassium phosphate buffer pH 7.4, 0.2 M urea at 25 °C. The loss of hyper-reactive cysteines due to reaction with GSSG was estimated by the titration of the –SH group with DTNB. The reactivity of free cysteines with GSSG was evaluated as reported in our previous study [16,17].

### 4.4. Reactivity of rRNase toward Several Disulfides and Thiol Reagents

The reactivity of the sulfhydryl groups of the rRNase (1.25 µM final concentration) toward DTNB (20 µM) was evaluated in a continuous spectrophotometric assay at 412 nm where TNBS^–^ absorbs (ε_M_ TNBS^–^ = 11800 M^−1^ cm^−1^ at pH 5.0). The first-order kinetic constants were evaluated on the basis of t_1/2_ at different DTNB concentrations. The reactivity of the rRNase (1.25 µM) toward homocystine (0.4 mM), cystine (0.2 mM), and cystamine (0.2 mM) was determined in 10 mM potassium phosphate buffer, pH 7.4, 0.2 M urea. At fixed times, aliquots were placed in 0.1 M acetate buffer, pH 5.0, 0.2 M urea and the disappearance of the reactive cysteines of the rRNase was determined using DTNB as the titrant (25 °C).

The reactivity toward CDNB was evaluated using continuous spectrophotometry at 340 nm where the Cys-DNB adduct absorbs (ԑ_M_ = 9600 M^−1^ cm^−1^) [16]. The rRNase (1.25 µM) was reacted with 0.4 mM CDNB in 0.1 M potassium phosphate buffer, pH 7.4, 0.2 M urea (25 °C). A slight turbidity due to the CDNB-modified enzyme was subtracted by each determination.

The reaction of the rRNase (1.25 µM) toward NBD-Cl (20 µM) was determined spectrophotometrically at 419 nm, where the Cys-NBD adduct absorbs (ԑ_M_ = 13000 M^−1^ cm^−1^) [32], in 0.1 M potassium phosphate buffer, pH 7.4, 0.2 M urea (25 °C).

Second-order kinetic constants of the reaction between free GSH (0.1 mM) and homocystine(0.4 mM) were determined by using 0.1 M potassium phosphate buffer, pH 7.4, to determine the amount of homocysteine released as a consequence of the reaction at fixed times. Homocysteine was determined by adding NaOH (20 mM final concentration) to the mixture and after the reaction with 0.3 mM bromopyruvate. The corresponding product is a cyclic ketimine sulfur compound (cystathionine ketimine) absorbing at 296 nm (ԑ_M_ = 3200 M^−1^ cm^−1^) [33]. Second-order kinetic constants for the reaction of free cysteine toward GSSG and free GSH toward all other reagents were derived from our previous study [17]. GSH solutions were freshly prepared and the amount of GSSG was less than 1% as assayed by standard analytical procedures.

### 4.5. pK_a_ Determination

The average p*K*_a_ of cysteines of the rRNase (1.25 μM) was calculated in a solution of 0.02 M Britton–Robinson buffer (pH varying from 4.0 to 11.0) and 0.2 M urea. The reactivity of these cysteines (8 –SH/mole rRNase) was measured with DTNB (20 μM), NBD-Cl (20 μM), and CDNB (1 mM). Only in the case of DTNB, the reaction was followed using an SFA-12 Rapid Kinetics Accessory (Hi-Tech Scientific, Bradford-on-Avon, UK). Below pH 7.0, appropriate TNBS^–^ extinction coefficients at 412 nm were considered. Normalized rates were calculated from observed initial velocities normalized to maximum velocities calculated at full deprotonation. The initial velocities of the reaction of the rRNase with the compounds were recorded spectrophotometrically in a continuous assay. Finally, p*K*_a_ were calculated by a curve fitting analysis.

### 4.6. Circular Dichroism Spectroscopy

CD spectra of the native RNase and rRNase in the presence of 0.2 M or 8 M urea were measured at 1.25 μM protein concentration in 10 mM potassium phosphate buffer, pH 7.4, 25 °C. A control sample of rRNase (6.5 μM) was alkylated with 1 mM bromopyruvate at pH 7.4 in 0.2 M urea (25 °C). A test sample of rRNase (6.5 μM) was incubated with 0.4 mM GSSG for 10 s and then alkylated with 1 mM bromopyruvate at pH 7.4 in 0.2 M urea (25 °C). Both these samples were eluted onto Sephadex G-25 to clean samples from GSSG and bromopyruvate. CD spectra of these two samples were measured at 1.25 μM protein concentration in 10 mM potassium phosphate buffer, pH 7.4, 0.2 M urea at 25 °C. The setting panel of the spectropolarimeter Jasco J-715 (Easton, MD) was: slit 2 nm, sensibility 50 mdeg, range 250–205 nm, resolution 0.2 nm, using a quartz cuvette of 0.5 cm light path.

### 4.7. Effect of Urea Concentration on the Hyper-Reactivity

The effect of urea on hyper-reactivity of the rRNase was assayed using DTNB as a thiol reagent. In a typical experiment, the rRNase (1.25 μM) was incubated with 0.01 M potassium phosphate buffer, pH 7.4, in the presence of variable concentrations of urea (from 0.2 M to 8 M). After five minutes of incubation, the rate of reaction with DTNB (20 μM) was measured spectrophotometrically at 412 nm in 0.1 M acetate buffer, pH 5.0 (25 °C).

### 4.8. Mass Spectrometry Identification of Hyper-Reactive Cysteine

The rRNase (1.25 μM) was incubated with GSSG (0.4 mM) in 0.01 M potassium phosphate buffer, pH 7.4. The reaction was stopped after 10 s by adding 1 mM bromopyruvate which alkylated residual protein cysteines within 1–2 s. Then the sample was lyophilized. A reduced RNase solution (1.25 μM) was immediately alkylated with bromopyruvate and used as a control. Samples were resuspended in 0.1% trifluoroacetic acid (TFA) and desalted by reverse-phase HPLC on a Phenomenex Jupiter C4 column (250 mm × 2.0 mm, 300 Å pore size) with a linear gradient from 10% to 95% of solvent B (0.07% TFA in 95% acetonitrile) in 30 min, at a flow rate of 200 μL/min using Agilent Technologies 1100 HPLC (Agilent Technologies, Santa Clara, CA, USA). Protein fractions were collected and lyophilized.

Controlled pepsin hydrolysis was carried out by dissolving the samples in 5% formic acid, pH 2.5 and adding pepsin at an enzyme to substrate ratio of 1:50 *w*/*w* at 37 °C for 2 h. Samples were then lyophilized and resuspended in 0.2% formic acid. Peptic hydrolysis was controlled by MALDI-MS analyses of the resulting peptide mixture and the samples were then directly analyzed by nanoLC/MS-MS on an LTQ-XL Orbitrap mass spectrometer equipped with a nanoHPLC (ThermoFisher, Waltham, MA, USA). Peptides containing modified cysteine residues were selected using the ion-extraction chromatograms of the corresponding multiply charged ions and the assignments were confirmed by manual inspection of their fragmentation spectra.

### 4.9. Statistical and Graphical Analysis

Data are represented as means ± standard deviation (S.D.). Data were obtained from independent experiments (from three to ten) performed in different days by the same operators using the same instruments. Statistical significance of the differences between second-order kinetic constants of the rRNase and free Cys/free GSH toward reagents was analyzed by *t*-tests. The same statistical analysis was applied for p*K*_a_ values. *p*-value < 0.05 was considered statistically significant. Statistical analyses were performed using the computer software package, MedCalc (Mariakerke, Belgium). The propagation of uncertainties for the quotients ‘enhanced reactivity’ was analyzed according to the classical statistical methods [20]. The equations used to fit kinetic data are:

Hyperbolic curve:
$y=\frac{vx}{K_{s}+x}$
(1)p*K*_a_ curve:
$y=\frac{10^{pH-pK_{a}}}{1+10^{pH-pK_{a}}}$
(2)Two-phase decay:
$y=y_{min}+\left(y_{max}-y_{min}\right)\left[ae^{-k_{1}x}+\left(1-a\right)e^{-k_{2}x}\right]$
(3)Sigmoidal curve:
$y=\frac{vx^{h}}{K_{s}+x^{h}}$
(4)One-phase decay:$y=y_{min}+\left(y_{max}-y_{min}\right)e^{-kx}$.(5)

The graphic and results visualization were obtained using GraphPad Prism software v5.0 (La Jolla, CA, USA).

## Figures and Tables

**Table 1:** Reactivity of rRNase cysteines toward disulfides and thiol reagents. Second-order kinetic constants *k* (M^−1^ s^−1^) for the reaction of cysteines of the rRNase, free Cys, and free GSH with natural disulfides and thiol reagents calculated at pH 7.4 and 25 °C.

	Free Cys	Free GSH	Reduced RNase A					
	*k* (M^−1^ s^−1^) ^e^	*k* (M^−1^ s^−1^) ^e^	*k* (M^−1^ s^−1^) ^e^	–SH/ mole rRNase	E.R. ^f^	*k* (M^−1^ s^−1^) ^e^	–SH/ mole rRNase	E.R. ^f^
GSSG	0.7 ± 0.1 ^a^ 0.2 ^b^	-	700 ± 40	1	1000 ± 150 3500	9 ± 1	5	13 ± 2 45
GSSG (8 M Urea)	0.7 ± 0.1 ^a^ 0.2 ^b^	-	0.8 ± 0.1 ^c^	6	1.1 ± 0.24	-	-	-
Cystamine	-	55 ± 2	250 ± 80	3	4.5 ± 1.4	-	-	-
Cystine	-	12 ± 1	53 ± 6	6	4.4 ± 0.6	-	-	-
Homocystine	-	0.6 ± 0.1	4 ± 1	8	7 ± 2	-	-	-
DTNB ^d^	-	20 ± 1	2500 ± 100	8	125 ± 8	-	-	-
CDNB	-	0.07 ± 0.01	0.9 ± 0.1	8	13 ± 2	-	-	-
NBD-Cl	-	8 ± 1	18 ± 2	8	2.3 ± 0.4	-	-	-

^a^ Experimental value for free Cys (p*K*_a_ = 8.53) [18]; ^b^ theoretical value for unperturbed protein cysteine (p*K*_a_ = 9.1) [19]; ^c^ differences between second-order kinetic constants were not significant; ^d^ experimental condition, pH 5.0 and 25 °C; ^e^ second-order kinetic constants are the average ± SD from five independent experiments; ^f^ enhanced reactivity. The error derived from the propagation of uncertainties (see Materials and Methods).

## Abbreviations

RNase	Ribonuclease
GSH	glutathione (reduced form)
GSSG	oxidized glutathione
rRNase	reduced ribonuclease
PDI	protein disulfide isomerase
BPTI	bovine pancreatic trypsin inhibitor
DTT	Dithiothreitol
DTNB	5,5′-dithiobis-(2-nitrobenzoic acid)
TNBS^-^	5-thio-2-nitrobenzoate
CDNB	1-chloro-2,4-dinitrobenzene
NBD-Cl	4-chloro-7-nitrobenzofurazan
CD	circular dichroism
EDTA	ethylendiaminetetraacetic acid
TFA	trifluoroacetic acid

## References

[1] Arolas J.L., Aviles F.X., Chang J.Y., Ventura S. (2006). Folding of small disulfide-rich proteins: Clarifying the puzzle. Trends Biochem. Sci..

[2] Dobson C.M. (2006). Protein Aggregation and its consequences for human diseases. Prot. Pept. Lett..

[3] Bechtel T.J., Weerapana E. (2017). From structure to redox: The diverse functional roles of disulfides and implications in disease. Proteomics.

[4] Stefani M., Dobson C.M. (2003). Protein aggregation and aggregate toxicity: New insights into protein folding, misfolding diseases and biological evolution. J. Mol. Med..

[5] Mossuto M.F. (2013). Disulfide bonding in neurodegenerative misfolding diseases. Int. J. Cell Biol..

[6] Dobson C.M. (2001). The structural basis of protein folding and its links with human disease. Philos. Trans. R. Soc. Lond. B Biol. Sci..

[7] Anfinsen C.B., Haber E., Sela M., White F.H. (1961). The kinetics of formation of native ribonuclease during oxidation of the reduced polypeptide chain. Proc. Natl. Acad. Sci. USA.

[8] Vinci F., Ruoppolo M., Pucci P., Freedman R.B., Marino G. (2000). Early intermediates in the PDI-assisted folding of ribonuclease A. Protein Sci..

[9] Wedemeyer W.J., Welker E., Narayan M., Scheraga H.A. (2000). Disulfide bonds and protein folding. Biochemistry.

[10] Rothwarf D.M., Li Y.-J., Scheraga H.A. (1998). Regeneration of bovine pancreatic ribonuclease A: Identification of two native like three-disulfide intermediates involved in separate pathways. Biochemistry.

[11] Rothwarf D.M., Li Y.-J., Scheraga H.A. (1998). Regeneration of bovine pancreatic ribonuclease A: Detailed kinetic analysis of two independent folding pathways. Biochemistry.

[12] Zito E., Chin K.T., Blais J., Harding H.P., Ron D. (2010). ERO1-beta, a pancreas-specific disulfide oxidase, promotes insulin biogenesis and glucose homeostasis. J. Cell. Biol..

[13] Lyles M.M., Gilbert H.F. (1991). Catalysis of the oxidative folding of ribonuclease A by protein disulfide isomerase: Pre-steady-state kinetics and the utilization of the oxidizing equivalents of the isomerase. Biochemistry.

[14] Wearne S.J., Creighton S.J. (1988). Further experimental studies of the disulfide folding transition of ribonuclease A. Proteins.

[15] Dadlez M., Kim P.S. (1996). Rapid formation of the native 14-38 disulfide bond in the early stages of BPTI folding. Biochemistry.

[16] Bocedi A., Fabrini R., Pedersen J.Z., Federici G., Iavarone F., Martelli C., Castagnola M., Ricci G. (2016). The extreme hyper-reactivity of selected cysteines drives hierarchical disulfide bond formation in serum albumin. FEBS J..

[17] Bocedi A., Cattani G., Martelli C., Cozzolino F., Castagnola M., Pucci P., Ricci G. (2018). The extreme hyper-reactivity of Cys94 in lysozyme avoids its amorphous aggregation. Sci. Rep..

[18] Torchinskii Y.M., Dixon H.B.F. (2013). Sulfhydryl and Disulfide Groups of Proteins.

[19] Harris T.K., Turner G.J. (2002). Structural basis of perturbed p*K*_a_ values of catalytic groups in enzyme active sites. IUBMB Life.

[20] Taylor J.R. (1997). An Introduction to Error Analysis.

[21] Chatani E., Hayashi R., Moriyama H., Ueki T. (2002). Conformational strictness required for maximum activity and stability of bovine pancreatic ribonuclease A as revealed by crystallographic study of three Phe120 mutants at 1.4 Å resolution. Protein Sci..

[22] Pettersen E.F., Goddard T.D., Huang C.C., Couch G.S., Greenblatt D.M., Meng E.C., Ferrin T.E. (2004). UCSF Chimera—A visualization system for exploratory research and analysis. J. Comput. Chem..

[23] Marino S.M., Vadim N., Gladyshev V.N. (2012). Analysis and Functional Prediction of Reactive Cysteine Residues. J. Biol. Chem..

[24] Porter C.M., Miller B.G. (2012). Cooperativity in monomeric enzymes with single ligand-binding sites. Bioorg. Chem..

[25] Xu X., Scheraga H.A. (1998). Kinetic folding pathway of a three-disulfide mutant of bovine pancreatic ribonuclease A missing the [40–95] disulfide bond. Biochemistry.

[26] Ruoppolo M., Vinci F., Klink T.A., Raines R.T., Marino G. (2000). Contribution of individual disulfide bonds to the oxidative folding of ribonuclease A. Biochemistry.

[27] Cuozzo J.W., Kaiser C.A. (1999). Competition between glutathione and protein thiols for disulphide-bond formation. Nat Cell Biol..

[28] Bass R., Ruddock L.W., Klappa P., Freedman R.B. (2004). A major fraction of endoplasmic reticulum-located glutathione is present as mixed disulfides with protein. J. Biol. Chem..

[29] Fraga H., Pujols J., Gil-Garcia M., Roque A., Bernardo-Seisdedos G., Santambrogio C., Bech-Serra J.J., Canals F., Bernadó P., Grandori R. (2017). Disulfide driven folding for a conditionally disordered protein. Sci. Rep..

[30] Pace C.N., Vajdos F., Fee L., Grimsley G., Gray T. (1995). How to measure and prediction the molar absorption coefficient of a protein. Protein Sci..

[31] Chakravarthi S., Jessop C.E., Bulleid N.J. (2006). The role of glutathione in disulphide bond formation and endoplasmatic-reticulum-generated oxidative stress. EMBO Rep..

[32] Birkett D.J., Price N.C., Radda G.K., Salmon A.G. (1970). The reactivity of SH groups with a fluorogenic reagent. FEBS Lett..

[33] Ricci G., Santoro L., Achilli M., Matarese R.M., Nardini M., Cavallini D. (1983). Similarity of the oxidation products of L-cystathionine by L-amino acid oxidase to those excreted by cystathioninuric patients. J. Biol. Chem..

